# Magnetic Resonance Imaging of the Small Bowel in Crohn's Disease: A Systematic Review and Meta-Analysis

**DOI:** 10.1155/2016/7857352

**Published:** 2016-04-21

**Authors:** Osman Ahmed, David Mario Rodrigues, Geoffrey C. Nguyen

**Affiliations:** Mount Sinai Hospital Centre for Inflammatory Bowel Disease, University of Toronto, Toronto, ON, Canada M5G 1X5

## Abstract

*Introduction*. Crohn's disease is most commonly found in the terminal ileum and colonic region. Magnetic resonance has become a useful modality for assessing small bowel activity. In this study, we performed a systematic review and meta-analysis on the use of MR in detecting small bowel activity as well as extramural complications in Crohn's patients.* Methods*. Two independent reviewers sorted through articles until October 2, 2014. We included both studies providing raw data for pooling and studies without raw data. Sensitivity, specificity, likelihood ratios, and 95% confidence intervals were calculated for each study.* Results*. There were 27 included studies, of which 19 were included in the pooled analysis. Pooled analysis of the 19 studies (1020 patients) with raw data revealed a sensitivity of 0.88 (95% CI 0.86 to 0.91) and specificity was 0.88 (95% CI 0.84 to 0.91). In regard to detecting stenosis, pooled sensitivity was 0.65 (95% CI 0.53 to 0.76) and specificity was 0.93 (95% CI 0.89 to 0.96).* Conclusion*. MR imaging provides a reliable alternative in detecting small bowel activity in patients with Crohn's disease. Its advantages include high diagnostic accuracy and no radiation exposure while its disadvantages include high cost and limited availability.

## 1. Introduction

Crohn's disease is an inflammatory bowel disease that may present with systemic symptoms such as fever, fatigue, and weight loss, as well as abdominal symptoms including pain and diarrhea [1]. Unlike ulcerative colitis, Crohn's disease can manifest anywhere in the gastrointestinal tract, though it most commonly affects the terminal ileum and colon. It is estimated that almost 50% of patients with Crohn's disease will have involvement of the small bowel, and up to 30% will have small bowel involvement only [2]. Crohn's disease is most often diagnosed by a combination of clinical features, endoscopy, and histopathology. Although the exact pathogenesis is still unclear, the disease manifests itself endoscopically as focal ulcerations with skip lesions (normal appearing bowel along with areas of inflammation) [1].

Recently, there has been an increase in the use of imaging modalities in assisting the diagnosis of Crohn's disease as well as assessing disease severity. Conventional enteroclysis, ultrasound (US), computed tomography (CT), and magnetic resonance (MR) imaging have all been used to detect inflammation in the bowel [3, 4]. The use of imaging for diagnosing small bowel activity has become even more relevant since traditional methods of diagnosis (e.g., endoscopy) are not able to visualize the small bowel reliably, and up to 10% of patients will have small bowel involvement not amenable to visualization by endoscopy [1]. Additionally, endoscopic methods of assessment carry the risk of procedural complications and can cause patient discomfort [5].

Multiple studies have investigated the use of imaging to diagnose small bowel activity in Crohn's patients. Although the use of conventional enteroclysis and CT have shown good diagnostic accuracy, they are limited by exposure to ionizing radiation [6]. Ultrasound is a nonradiating form of imaging but is limited because image quality is dependent on technician expertise [7].

Magnetic resonance has become a useful modality for assessing small bowel activity in Crohn's disease, with multiple studies showing great sensitivity and specificity. The use of enteral contrast agents using MR enterography protocols has allowed for better distention as well as visualization of the small bowel [8]. Previous studies and reviews have looked at the use of MR in Crohn's disease; however these studies were limited in only assessing small bowel activity or extraluminal complications [9, 10]. Multiple new studies have since emerged looking at this field. In this study, we performed a systematic review and meta-analysis on the use of MR in detecting small bowel activity as well as intra- and extraluminal complications in Crohn's patients. We also determined whether the use of MR enteroclysis, a recent method of administrating contrast, yields any advantages over conventional MR enterography.

## 2. Materials and Methods

### 2.1. Search Strategy

We performed a comprehensive search strategy with the use of electronic databases including MEDLINE, EMBASE, CINAHL, and Cochrane Central Register of Controlled Trials. All relevant articles published until October 2, 2014, were included. Our search strategy included individual and combinations of relevant terms including “Crohn's”, “inflammatory bowel disease”, and “magnetic resonance” (see Supplementary Table 1 in Supplementary Material available online at http://dx.doi.org/10.1155/2016/7857352 for comprehensive search terms used). References of selected articles and previously published review articles were also manually searched to identify relevant studies.

### 2.2. Study Selection and Data Extraction

Our inclusion criterion was any study that compared the use of magnetic resonance to diagnose small bowel activity in Crohn's disease. We used as a reference standard surgery, ileocolonoscopy, and/or histopathology individually or as components of a global consensus. Small bowel was defined as any region distal to the pylorus up until the area proximal to the ileocecal junction. Authors of studies that combined small and large bowel data were contacted in order to obtain data for small bowel only. If we received no response, the study was excluded from the review. Studies which did not provide per-patient raw data (in terms of true positive, true negative, false positive, and false negative values) for the small bowel were also contacted for that information. If we received no response from the authors, they were still included in the review (including studies with only per-segment raw data) but excluded from the meta-analysis. Studies that comprised pediatric populations, were non-English, or used a reference standard that did not include surgery, ileocolonoscopy, or histopathology were excluded. Abstracts, conference presentations, and posters were also excluded.

All retrieved studies were sorted independently by two reviewers (Osman Ahmed and David Mario Rodrigues). Any disagreements were resolved either by consensus or by a third reviewer. Data from all selected studies was extracted independently by the same two reviewers (Osman Ahmed and David Mario Rodrigues). They followed a data extraction form that was created* a priori* and included study characteristics (year, country, age, gender, number of patients, type of study, reference standard, patient population, and location studied) and imaging characteristics (enterography versus enteroclysis, magnetic field strength, oral and intravenous contrast, bowel preparation, radiologist's experience, and time interval between MR and reference standard). Finally, specific values were extracted or calculated for studies that provided per-patient raw data (true positive, true negative, false positive, and false negative). For studies that included per-patient values for different segments of the small bowel, only the most distal part of the small bowel was included as this is the area most commonly affected by Crohn's disease.

Assessment of risk of bias, quality, and applicability was performed by using the Quality Assessment of Diagnostic Accuracy Studies (QUADAS-II) tool developed for diagnostic studies [11]. Studies with scores greater than 9 were categorized as low-risk.

### 2.3. Statistical Analysis

For studies that did not provide raw data, a summary of the results was presented for small bowel activity using sensitivity and specificity values. For studies providing raw data, 2 × 2 contingency tables were created using the following variables (true positive, true negative, false positive, and false negative). Statistical analysis was performed with use of Meta-DiSc version 1.4 (J. Zamora, A. Muriel, and V. Abraira Meta-DiSc for Windows, XI Cochrane Colloquium, Barcelona, 2003) software. Sensitivity, specificity, likelihood ratios, and 95% confidence intervals were calculated for each study. Figures for forest plots and summary receiver-operating characteristic (ROC) curves were also constructed using the software, while the area under the curve (AUC) was calculated. For pooled analysis, 0.5 was added to all cells that contained a value of 0 in order to include all studies in the analysis. Heterogeneity was assessed using the *I* ^2^ test.

## 3. Results

The initial search yielded 3981 studies. These studies were initially sorted by title yielding 332 studies, which were then limited to 29 studies based on abstract review. After retrieving the full articles and contacting authors for missing information, only 27 studies met the inclusion criteria (Supplementary Figure 1). Of the 27 included studies, 19 provided sufficient raw data to be included in the pooled analysis; eight studies provided only summaries of the results [12–38]. All studies had a QUADAS-II score equal to or greater than 9, indicating low-risk.

### 3.1. Study Characteristics

Individual study characteristics are reported in Tables 1 and 2. A total of 19 studies with 1020 patients were included in the meta-analysis. Eight studies with a total of 650 patients were only included in the systematic review (Table 3) [12–38].

There were 17 prospective studies [12, 15–17, 19, 21–23, 26–29, 31, 32, 34, 35, 38], 4 retrospective studies [25, 30, 33, 37], and one study [36] that had both retrospective and prospective components. An additional 5 studies were unclear in regard to the type of study [13, 14, 18, 20, 24]. In regard to patient population, 13 out of the 27 studies involved patients with established Crohn's disease [12, 14–17, 24, 26–30, 36–38]; 3 had only suspected CD [20, 21, 35]; and 11 had either suspected or established CD [13, 18, 19, 22, 23, 25, 28, 31–34]. Most prospective studies used consecutive patients to limit selection bias. Study characteristics for all studies are summarized in Table 1.

In regard to imaging characteristics, most studies used a magnetic field strength of 1.5 T, with 2 studies using 1.0 T [12, 23] and 2 studies using 3.0 T [34, 35]. Three studies did not mention the magnetic field strength used [13, 31, 37]. Six of the 27 studies used enteroclysis as a method of introducing oral contrast [13, 14, 16, 18, 20, 31], while 19 studies used standard enterography. One study used both enterography and enteroclysis [19], while 1 study did not mention how oral contrast was given [15]; neither of these studies were included in the meta-analysis. Radiologist experience with abdominal MR and time interval between MR and the reference standard varied widely between the studies (Table 2).

In regard to disease activity, unless otherwise specified below, we considered positive small bowel activity to be when the individual study considered the disease active (no specific parameters were used). Maccioni et al. [17] provided raw data for both T1-weighted and T2-weighted imaging. We chose to include T2-weighted results as they have been shown previously to be more accurate for small bowel activity [39]. Oto et al. selected for patients with active disease and thus had no results for specificity [30]. Because Seiderer et al. used an anterograde endoscopic approach, the jejunum was used for analysis as very few patients had their terminal ileum intubated [20]. For Adamek et al., we used histopathology as the reference standard rather than ileocolonoscopy (both were provided) [34]. Alternatively, for Siddiki et al., we used ileocolonoscopy as the reference standard since not as many patients had histopathology results [21]. For Kumar et al., we used bowel thickening as representative of small bowel activity [37]. Jensen et al. published two studies in 2011. Because there was no overlap in the study populations, both were included in our meta-analysis [28, 29].

#### 3.1.1. Nonpooled Studies Summary

Of the 8 studies which were not included in the pooled analysis, by far the largest was Grand et al. with 310 patients (out of 650 total patients) [33]. Of these, 162 underwent MR and endoscopy within 30 days and the per-patient analysis for the distal ileum revealed a sensitivity of 85% and a specificity of 79% in diagnosing Crohn's disease activity. The results of the remaining studies are summarized in Table 3.

### 3.2. Per-Patient Pooled Analysis

**Figure 1:**
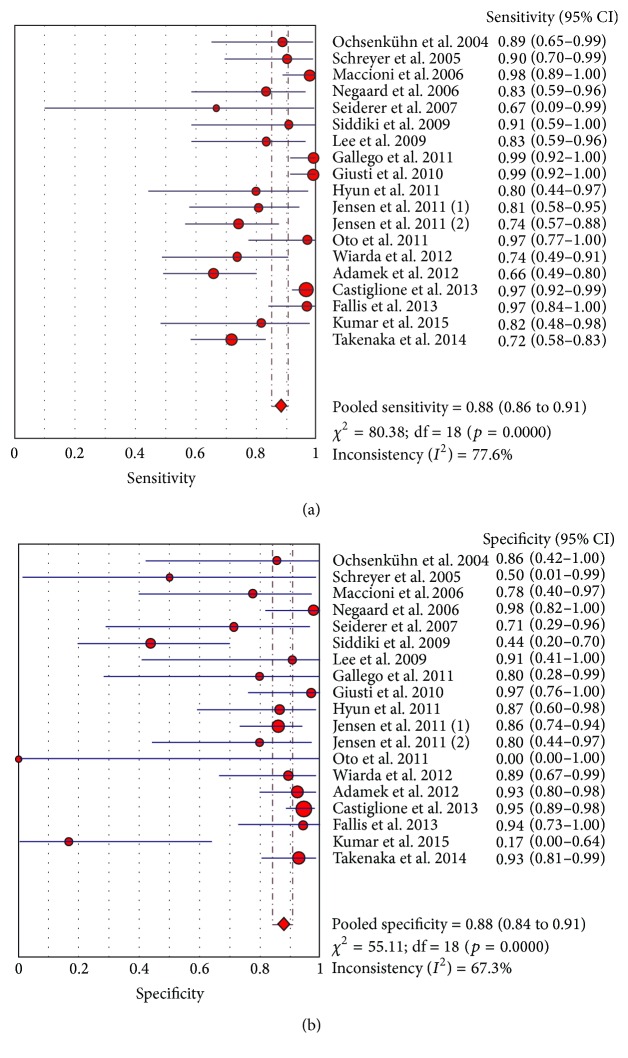
Sensitivity and specificity for active Crohn's disease (all studies).

Forest plots for the sensitivity, specificity, and sROC for the use of MR in diagnosing small bowel Crohn's disease activity are presented (Figure 1, Supplementary Figure 2). Pooled analysis of the 19 studies with raw data revealed a sensitivity of 0.88 (95% CI 0.86 to 0.91) with a heterogeneity of *χ* ^2^ = 80.38 and *I* ^2^ of 77.6% (Figure 1). The pooled specificity was 0.88 (95% CI 0.84 to 0.91) with a heterogeneity of *χ* ^2^ = 55.11 and *I* ^2^ of 67.3% (Figure 1). Using a random effects model, the positive likelihood ratio was 5.2 (95% CI 2.62 to 10.29) and the negative likelihood ratio was 0.17 (95% CI 0.11 to 0.27) (Supplementary Figure 3). Using Moses' constant linear model, we were able to construct an sROC with an AUC of 0.93 (Supplementary Figure 2). As expected, most values reside in the left upper corner, suggesting high sensitivity and specificity.

### 3.3. Subgroup Analysis

**Figure 2:**
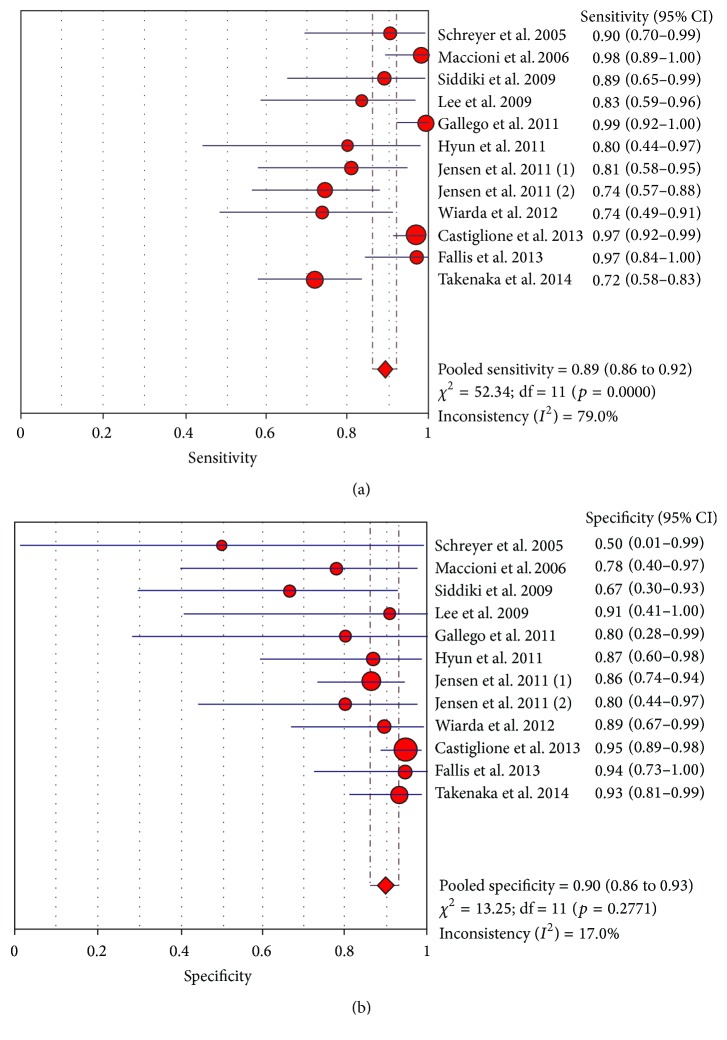
Sensitivity and specificity for active Crohn's disease (prospective studies only).

**Figure 3:**
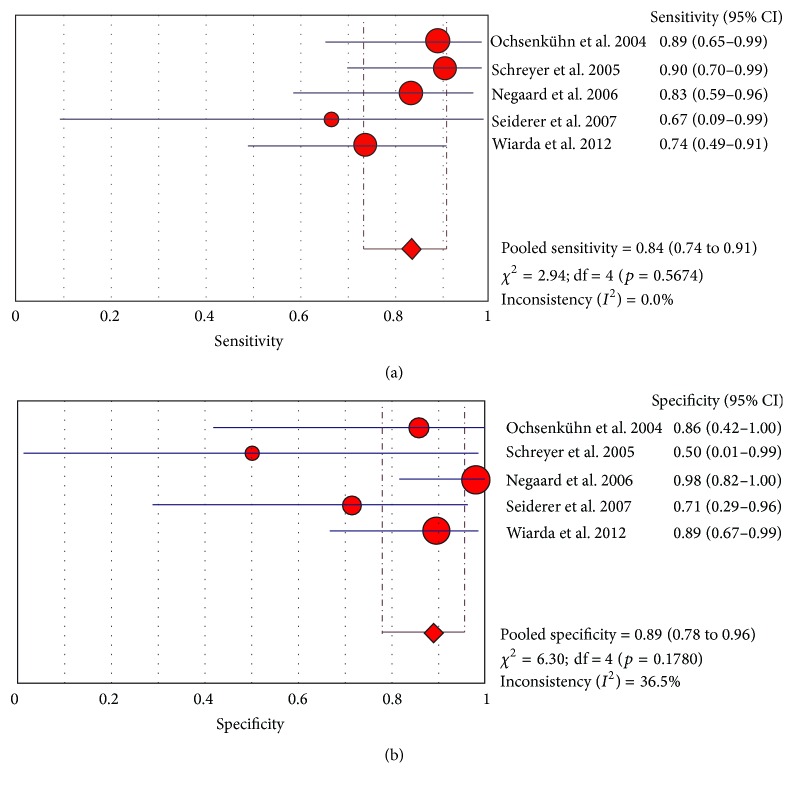
Sensitivity and specificity for active Crohn's disease (enteroclysis studies only).

Analysis of only prospective studies revealed a pooled sensitivity of 0.89 (95% CI 0.86 to 0.92) and a pooled specificity of 0.90 (95% CI of 0.86 to 0.93) (Figure 2). Five studies used enteroclysis as the method of administrating oral contrast [14, 16, 18, 20, 31]. Pooled analysis of these 5 studies gave a sensitivity of 0.84 (95% CI of 0.74 to 0.91) and a pooled specificity of 0.89 (95% CI of 0.78 to 0.96) as demonstrated in the forest plots (Figure 3).

### 3.4. Extramural Complications

**Figure 4:**
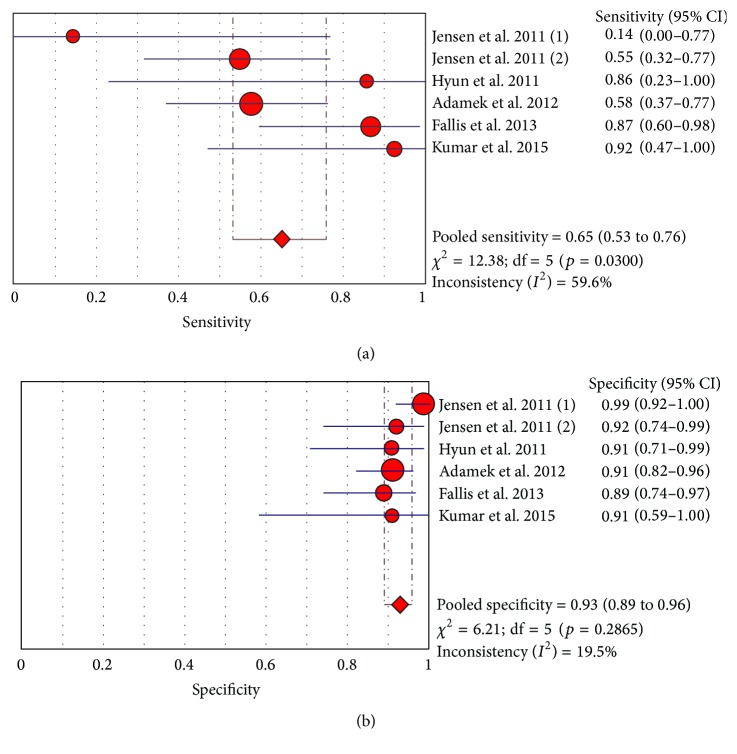
Sensitivity and specificity for stenosis in Crohn's disease (all studies).

We identified three extramural complications* a priori* (stenosis, fistula, and abscess). However, only two studies (Fallis et al. and Kumar et al.) [36, 37] provided raw data for analysis for fistulas and abscesses. Consequently, no pooled analysis was done for these complications. We conducted a pooled analysis and constructed forest plots for 6 studies that provided data for stenosis (Figure 4) [9, 27–29, 34, 36, 37]. Pooled sensitivity was 0.65 (95% CI 0.53 to 0.76) and pooled specificity was 0.93 (95% CI 0.89 to 0.96).

## 4. Discussion

Our study represents the largest systematic review (in terms of patients and number of studies) of MR imaging for the detection of small bowel activity in Crohn's disease. Like previous studies and reviews, we demonstrate that MR imaging possesses high sensitivity and specificity in detecting small bowel activity [3, 9, 10]. Along with a relatively high positive likelihood ratio and relatively low negative likelihood ratio, it can be used in combination with pretest probabilities to determine small bowel activity in the appropriate clinical setting. The results from this meta-analysis are similar to those previously reported [9]. Of note, many of the studies differ from those included in previous reviews, not only because we focused only on the small bowel, but also because we included both studies with per-patient and per-segment analysis (though only per-patient analysis was pooled). Additionally, differences in response rates in regard to contacting authors likely explain the discrepancy in studies included in our review as compared to others.

One of the many research areas in MR imaging is the use of enteroclysis in inflammatory bowel disease. Enteroclysis has been proposed to provide better small bowel distension because the contrast is provided directly through nasojejunal intubation rather than orally [40]. The limitations of enteroclysis are that it is not as widely available, requires fluoroscopic insertion of a nasojejunal tube (thus exposing patients to radiation), and is less well tolerated by patients [41]. A subgroup analysis of studies using enteroclysis did not demonstrate higher sensitivity and specificity. Similarly, Negaard et al. directly compared use of oral contrast and enteroclysis and also did not find any significant difference [19]. One explanation for the lack of increment benefit of enteroclysis is because small bowel Crohn's disease usually affects the terminal ileum distally, rather than proximally, where the advantages of enteroclysis are more apparent. Overall, enteroclysis has not yet been shown to have any significant difference in diagnostic accuracy, and its role in Crohn's patients is still uncertain. Further study is warranted in determining potential benefits of enteroclysis in Crohn's disease proximal to the ileum.

In regard to intra- and extraluminal complications such as fistulas, abscesses, and stenosis, MR has been theorized to be the gold standard, since ileocolonoscopy can only assess luminal disease, and surgery is too invasive and not a feasible diagnostic modality. Our analysis revealed fairly high specificity in detecting stenosis, but only moderate sensitivity. However, our analysis was limited due to the small number of studies included. Previous studies looking at both small and large bowel have shown relatively high detection rates for stenosis [26, 42]. Nevertheless, a meta-analysis by Qiu et al. showed that CT imaging may be better at detecting fistulas and stenosis. However, the results were not statistically significant, and the sensitivities and specificities were comparable to more recently published studies [43, 44]. No differences were noted in detecting abscesses. The results for pooled analysis by Qiu et al. for stenosis revealed similar numbers to our study (sensitivity 65.3%, specificity 94.4%).

Current European guidelines recommend MR, US, and CT enterography or enteroclysis for the detection of intestinal involvement and penetrating lesions in CD. Additionally, the use of small bowel follow-through or small bowel enteroclysis is acceptable for detection of stenosis. They are also the recommended techniques for detection of extramural complications of CD [1, 45]. American guidelines are less specific but do include the use of MR, amongst a multitude of other imaging modalities, to delineate and discriminate intra-abdominal masses/abscesses and in the evaluation of small bowel pathology in patients with CD [46].

One of the difficulties in replacing the gold standard with MR imaging is the lack of standardization of the imaging signs suggestive of active disease, especially with the growing number of sequences available. Previous studies have demonstrated that the most accurate signs of inflammation for MR were wall enhancement, mucosal lesions, and wall T2 hyperintensity [39, 47]. In one study, Maccioni et al. looked at the difference using T1- versus T2-weighted imaging. They found that T2-weighted images provided greater sensitivity and specificity in diagnosing ileal lesions [17]. Additionally, a study by Udayasankar et al. found similar results in both the small and large bowel [48]. Previous studies have also found that sequences using diffusion-weighted imaging had high sensitivity and specificity [30, 49, 50]. Recently, there have been development of validated scoring systems including the MaRIA (Magnetic Resonance Index of Activity) score for assessment of disease activity and severity, the Lemann score, or the Crohn's Disease Digestive Damage Score, which takes into account many factors (clinical, endoscopic, and imaging findings) and attempts to measure cumulative damage [51, 52].

An important consideration is whether the results of MR imaging change clinician management. A study by Mendoza et al. showed that MR helped in decision-making in more than half of patients, especially those involving the use of biological therapies and surgery [53]. Messaris et al. showed that 69% of patients had changes to medical and/or surgical management after clinicians were given MR imaging results [54]. Similar results have shown that MR findings influence surgical approaches to managing Crohn's patients [55].

Some of the limitations of our study include the varied length of time between the reference standard and MR imaging. Since clinical activity can change quite drastically, especially with the use of medications, some of the results might have been inaccurate in determining disease activity. Secondly, since we used per-patient data, we were not able to differentiate severity in regard to determining small bowel activity. Other studies have suggested that MRI has good correlation with Crohn's severity indices [56]. Similarly, we were unable to perform per-segment analysis which might have led to an overestimation of the accuracy of MR imaging. This is likely due to the use of endoscopy as a reference standard and its inadequacy in assessing more proximal small bowel. Other limitations include the fact that we were only able to analyse one complication (stenosis), and that others (such as abscess and fistulas) are not well visualized on endoscopy. Similarly, due to the small number of studies, we were not able to determine whether more advanced MR (such as MR with 3.0 T magnetic field strength) had any additional benefit. Finally, the large heterogeneity amongst the studies, including reference standards, radiologists experience, and results, suggests that more definitive studies might still be required. Sources of heterogeneity include inclusion of studies of different sample size and different criteria for disease activity, as well as inclusion of studies using different MR enterography/enteroclysis protocols (including different magnetic field strengths, oral contrast, and radiologist experience).

In regard to other modalities of imaging, many studies assessed the use of ultrasound and computed tomography. The benefit of ultrasound is that it does not involve ionizing radiation and is relatively inexpensive [3]. Previous studies assessing ultrasound have demonstrated high sensitivities and specificities. There is one large-scale trial comparing US and MR currently in progress: the UK-based MR Enterography or Ultrasound in Crohn's disease (METRIC) trial [3, 57]. The use of US is thought to be limited by operator-experience; however MR has also been shown to have interobserver variability. However, the use of scoring systems such as MaRIA has been proven to improve interobserver agreement [58]. Similarly, one meta-analysis has shown similar accuracy between CT and MR. CT has the benefit of being widely available and cost-effective. It, however, also carries the risk of ionizing radiation, especially amongst patients who might require multiple scans throughout the course of their life-long disease [43].

In conclusion, MR imaging provides a reliable alternative to ileocolonoscopy in detecting small bowel activity in patients with Crohn's disease. Its advantages include high diagnostic accuracy, favorable safety profile, and the ability to assess intra- and extraluminal complications. Its disadvantages include high cost and limited availability. Nevertheless, with the rapid expansion in MR accessibility, it will likely play a greater role in the future in both the diagnosis and management of patients with Crohn's disease.

## Supplementary Material

See Supplementary Material for comprehensive search strategy, flowchart, and figures for summary receiver operating characteristic curve (sROC), as well as positive and negative likelihood ratios.

## Figures and Tables

**Table 1:** Study characteristics.

Name	Year	Country	Number of patients (patients included)	Age (range)	Prospective/retrospective	Patient population	Reference standard	Location studied	Pooled analysis (raw data available)
Rieber et al. [13]	2000	Germany	194 (84)	NS	NS	Suspected or established CD	Ileocolonoscopy ± histopathology	Small bowel	No

Koh et al. [12]	2001	United Kingdom	30 (21)	37.6 (18–58)	Prospective	Established CD	Surgery ± ileocolonoscopy	Small and large bowel	No

Ochsenkühn et al. [14]	2004	Germany	29 (25)	31.5 (19–58)	NS	Established CD	Ileocolonoscopy ± histology	Small bowel	Yes

Pascu et al. [15]	2004	Germany	61 (37)	38 (19 to 84)	Prospective	Established CD	Ileocolonoscopy	Small and large bowel	No

Schreyer et al. [16]	2005	Germany	30 (23)	29 (18–65)	Prospective	Established CD	Ileocolonoscopy	Small and large bowel (terminal ileum for analysis)	Yes

Maccioni et al. [17]	2006	Italy	70 (59)	46.3 (18–76)	Prospective	Established CD	Ileocolonoscopy ± imaging	Small and large bowel (ileum for analysis)	Yes

Negaard et al. [18]	2006	Norway	60 (41)	33 (16–65)	NS	Suspected or established CD	Ileocolonoscopy ± surgery	Small bowel (terminal ileum for analysis)	Yes

Negaard et al. [19]	2007	Norway	48 (35)	39 (18–73)	Prospective	Suspected or established CD	Surgery, ileocolonoscopy ± histopathology ± capsule endoscopy	Small bowel	No

Seiderer et al. [20]	2007	Germany	10 (10)	33.9 (17–57)	NS	Suspected CD	Double-balloon enteroscopy (oral route)	Small bowel (jejunum for analysis)	Yes

Siddiki et al. [21]	2009	USA	33 (27)	39 (20–60)	Prospective	Suspected CD	Ileocolonoscopy ± histopathology	Small bowel	Yes

Lee et al. [22]	2009	South Korea	31 (23)	28.8 (18–44)	Prospective	Suspected or established CD	Ileocolonoscopy	Small bowel	Yes

Giusti et al. [24]	2010	Italy	70 (70)	27.8 (15–45)	NS	Established CD	Histopathology	Small bowel	Yes

Parisinos et al. [25]	2010	United Kingdom	342 (68)	36.8 (25–47)	Retrospective	Suspected or established CD	Surgery, ileocolonoscopy ± histopathology	Small bowel	No

Fiorino et al. [26]	2011	Italy	44 (44)	43 (19–61)	Prospective	Established CD	Ileocolonoscopy	Small and large bowel (ileum for analysis)	No

Gallego et al. [23]	2011	Spain	61 (61)	36.1 (14–65)	Prospective	Suspected or established CD	Ileocolonoscopy	Small bowel (ileum for analysis)	Yes

Hyun et al. [27]	2011	Japan	30 (25)	29.5 (24–38)	Prospective	Established CD	Ileocolonoscopy ± double-balloon enteroscopy (rectal route)	Small and large bowel (small bowel for analysis)	Yes

Jensen et al. [28]	2011	Denmark	93 (72)	30 (15–74)	Prospective	Suspected or established CD	Ileocolonoscopy ± surgery	Small bowel	Yes

Jensen et al. [29]	2011	Denmark	53 (45)	39 (18–76)	Prospective	Established CD	Ileocolonoscopy ± surgery	Small bowel	Yes

Oto et al. [30]	2011	USA	18 (18)	33.2 (20–53)	Retrospective	Established CD	Endoscopy ± histopathology	Small bowel	Yes

Wiarda et al. [31]	2012	Netherlands	41 (38)	36 (20–74)	Prospective	Suspected or established CD	Balloon-assisted enteroscopy and consensus	Small bowel	Yes

Friedrich et al. [32]	2012	Germany	79 (39)	27.8 (23–48)	Prospective	Suspected or established CD	Ileocolonoscopy	Small and large bowel (ileum for analysis)	No

Grand et al. [33]	2012	USA	310 (310)	45 (20–94)	Retrospective	Suspected or established CD	Ileocolonoscopy ± histopathology	Small and large bowel	No

Adamek et al. [34]	2012	Germany	104 (82)	39.8 (18–68)	Prospective	Suspected or established CD	Ileocolonoscopy + histopathology	Small bowel (terminal ileum for analysis)	Yes

Castiglione et al. [35]	2013	Italy	265 (234)	39	Prospective	Suspected CD	Ileocolonoscopy ± surgery	Small bowel	Yes

Fallis et al. [36]	2013	United Kingdom	51 (51)	41.3 (17–79)	Both	Established CD	Surgery	Small and large bowel (distal ileum for analysis)	Yes^1^

Takenaka et al. [38]	2014	Japan	100 (100)	31 (16–71)	Prospective	Established CD	Balloon-assisted enteroscopy (rectal route)	Small bowel (terminal ileum for analysis)	Yes

Kumar et al. [37]	2015	United Kingdom	17 (17)	30.8 (19–72)	Retrospective	Established CD	Surgery	Small bowel	Yes

^1^Provided by author.

**Table 2:** Imaging characteristics.

Name	Year	Enterography/ enteroclysis	Magnetic field strength	Type of coil	Bowel preparation	Intravenous contrast	Oral contrast	Blinded	Radiologist experience	Time interval between MR and RS
Rieber et al. [13]	2000	Enteroclysis	NS	NS	20 mg IV N-butyl scopolamine	Gadolinium-DTPA	800 mL barium sulfate solution and 1200 mL methyl cellulose solution	NS	NS	NS

Koh et al. [12]	2001	Enterography	1.0 T	NS	1 mg IM glucagon	0.1 mmol/kg gadodiamide	600 mL water	Yes	NS	Median 21 days

Ochsenkühn et al. [14]	2004	Enteroclysis	1.5 T	NS	30–60 mg IV butylscopolamine	0.1 mmol/kg gadolinium-DTPA	1500–2000 mL of a suspension (12 g iron-containing Ferristen) + 20 g methylcellulose (NJ)	Yes	Two experienced gastrointestinal radiologists	Median of 10 (3–13) weeks

Pascu et al. [15]	2004	NS	1.5 T	Body coils	NS	0.2 mmol/kg gadolinium-DTPA	NS	Yes	NS	Within 5 days

Schreyer et al. [16]	2005	Enteroclysis	1.5 T	Polarized 4-element phased-array body coil	40 mg IV N-butyl-scopolamine	0.2 mmol/kg gadolinium-DTPA	1000 mL water (25 g mannitol and 5 g carob seed)	Yes	NS	Within 1 week

Maccioni et al. [17]	2006	Enterography	1.5 T	Phased-array coil	NS	0.18 mmol/kg gadopentetate dimeglumine	600–900 mL ferumoxsil solution	Yes	>8 years of experience and resident	Within 15 days

Negaard et al. [18]	2006	Enteroclysis	1.5 T	Phased-array abdomen surface coil	20 mg IV scopolamine butylbromide	0.1 mmol/kg gadolinium-DTPA	1500–2000 mL polyethylene glycol	Yes	Two and 1 years of experience with MRE	Within 4 months

Negaard et al. [19]	2007	Both	1.5 T	Phased-array coils	20 mg IV scopolamine butylbromide	0.1 mmol/kg gadolinium-DTPA	1000 mL of 6% mannitol or 1500–2000 mL of polyethylene glycol solution (NJ)	Yes	>8- and >3-year experience	Within 3 months

Seiderer et al. [20]	2007	Enteroclysis	1.5 T	NS	40 mg IV butylscopolammonium bromide	0.1 mmol/kg gadolinium-DTPA	2500 mL 0.5% methylcellulose solution (NJ)	NS	Two board-certified radiologists	Within 6 weeks after MRE

Siddiki et al. [21]	2009	Enterography	1.5 T	16-channel torso array coil	0.5 mg IV glucagon	0.2 mmol/kg gadodiamide	1350 mL barium preparation	Yes	NS	Within 30 days

Lee et al. [22]	2009	Enterography	1.5 T	Two six-element, phased-array body coils	20 mg IV scopolamine-*N*-butyl bromide	15 mL of gadopentetate dimeglumine	1200 mL 3% sorbitol solution and 4000 mL polyethylene glycol solution	Yes	Six- and 10-year experience	Within 1 week

Giusti et al. [24]	2010	Enterography	1.5 T	Two phased-array coils	20 mg IV hyoscine-*N*-butylbromide	0.1 mL/kg 1.0 M gadolinium chelate	1500–2000 mL polyethylene glycol solution	Yes	Two with >10-year experience	NS

Parisinos et al. [25]	2010	Enterography	1.5 T	Two multichannel phased-array body coils	20 mg IV hyoscine-*N*-butylbromide	15 mL of gadolinium chelate	1500 mL of 2.5% mannitol and 0.5% locust bean gum solution	NS	NS	Within 8.5 to 112 days

Fiorino et al. [26]	2011	Enterography	1.5 T	Phased-array surface coil	0.5 mg of glucagon IV	Gadolinium	700 mL polyethylene glycol solution	Yes	Two with >8-year experience	Within 26 days (range 0–37)

Gallego et al. [23]	2011	Enterography	1.0 T	Multichannel-body coil	20–40 mg IV hyoscine bromide	0.1 mmol/kg gadopentetate dimeglumine	1500 mL polyethylene glycol solution	Yes	Two experienced radiologists	Within 15 days

Hyun et al. [27]	2011	Enterography	1.5 T	32-element body coil	20 mg IV scopolamine butylbromide	0.2 mL/kg gadolinium chelate	1000 mL–1500 mL polyethylene glycol solution	Yes	Two board-certified radiologists	Same day

Jensen et al. [28]	2011	Enterography	1.5 T	Five-element Syn-body coil	20 mg IV Hyoscinbutylbromide	0.1 mmol/kg gadodiamide	1000 mL 7.5% mannitol solution	Yes	Five with >4-year experience	Median 13 days

Jensen et al. [29]	2011	Enterography	1.5 T	Five-element Syn-body coil	20 mg IV Hyoscinbutylbromide	0.1 mmol/kg gadodiamide	1000 mL 7.5% mannitol solution	Yes	Five with >4-year experience	Median 11 days (51 days for surgery)

Oto et al. [30]	2011	Enterography	1.5 T	Four-channel phased-array body coil	1 mg IM glucagon	0.1 mmol/kg gadodiamide	1350 mL Volumen	NS	12 years of experience in body MRI	Median of 14 (0–62) days

Wiarda et al. [31]	2012	Enteroclysis	NS	NS	20 mg IV butylscopolamine bromide	0.1 mmol/kg of gadobutrol	1000–3000 mL 0.5% methylcellulose solution (ND)	Yes	>200 MRE studies	Median of 22 (4–112) days

Friedrich et al. [32]	2012	Enterography	1.5 T	Circular polarized 6-channel phased-array body coil	40 mg IV N-butyl scopolamine	0.1 mmol/kg gadolinium-DTPA	1000 mL water (25 g mannitol and 5 g carob seed) and 2000 mL water	Yes	Two with 6- and 7-year experience	Within 3 weeks

Grand et al. [33]	2012	Enterography	1.5 T	Eight-channel torso array coil or 4-channel surface coil	NS	0.1 mmol/kg gadopentetate dimeglumine	900 mL Volumen	Yes	>4-year experience	Within 90 days

Adamek et al. [34]	2012	Enterography	3.0 T	Two surface coils	40 mg IV hyoscine-*N*-butylbromide	Gadodiamide	1500 to 2000 mL mannitol solution	Yes	>5-year experience	Within 7 days

Castiglione et al. [35]	2013	Enterography	3.0 T	Two-paired phased-array body coils	20 mg IV N-butylscopolamine	0.2 mmol/kg gadopentetate dimeglumine	1500 mL polyethylene glycol solution	Yes	Two expert radiologists	NS

Fallis et al. [36]	2013	Enterography	1.5 T	Abdominal phased-array coils	20 mg IV hyoscine-*N*-butylbromide	0.2 mL/kg gadoterate meglumine	1200–1300 mL 3% mannitol solution	Yes	Dedicated gastrointestinal radiologist	Mean 10.8 (1–52) weeks

Takenaka et al. [38]	2014	Enterography	1.5 T	NS	20 mg IV scopolamine butylbromide	0.2 mL/kg gadolinium chelate	1000 mL polyethylene glycol	Yes	Two board-certified radiologists	Within 3 days

Kumar et al. [37]	2015	Enterography	NS	Eight-channel body coil	Hyoscine butylbromide	0.2 mL/kg gadolinium	250 mL 20% mannitol solution	NS	NS	Mean 77.4 days

NS: not specified; NJ: nasojejunal intubation; ND: nasoduodenal intubation.

**Table 3:** Results (of nonpooled studies).

Name	Year	Number of patients (number of patients included in analysis)	Results
Rieber et al. [13]	2000	194 (84)	Sensitivity and specificity of 95.2% and 92.6% in terminal ileum

Koh et al. [12]	2001	30 (21)	Sensitivity and specificity of 89% and 67% in terminal ileum

Pascu et al. [15]	2004	61 (37)	Per-segment sensitivity and specificity of 56% and 73% in terminal ileum

Negaard et al. [19]	2007	48 (35)	Sensitivity and specificity of 88% and 89% for MRI with OS, and 88% and 84% for MR enteroclysis

Parisinos et al. [25]	2010	342 (68)	Sensitivity and specificity of 85.1% and 85.71% in ileum

Fiorino et al. [26]	2011	44 (44)	Sensitivity and specificity of 93% and 81% in ileum

Friedrich et al. [32]	2012	79 (39)	Sensitivity and specificity of 72% and 87% in terminal ileum without rectal enema; 100% and 74% in terminal ileum with rectal enema

Grand et al. [33]	2012	310 (310)	Sensitivity and specificity of 85% and 79% in distal ileum
